# Identification reproducible microbiota biomarkers for the diagnosis of cirrhosis and hepatocellular carcinoma

**DOI:** 10.1186/s13568-023-01539-6

**Published:** 2023-03-21

**Authors:** Huarong Zhang, Junling Wu, Yijuan Liu, Yongbin Zeng, Zhiyu Jiang, Haidan Yan, Jie Lin, Weixin Zhou, Qishui Ou, Lu Ao

**Affiliations:** 1 Key Laboratory of Ministry of Education for Gastrointestinal Cancer, the School of Basic Medical Sciences, Fujian Medical University, Fuzhou, 350122 China; 2 Department of Bioinformatics, Fujian Key Laboratory of Medical Bioinformatics, School of Medical Technology and Engineering, Fujian Medical University, Fuzhou, 350122 China; 3 Department of Gastroenterology, The First Affiliated Hospital of Fujian Medical University, Fuzhou, 350005 China; 4 Department of Laboratory Medicine, Gene Diagnosis Research Center, The First Affiliated Hospital of Fujian Medical University, Fuzhou, 350005 China; 5 Fujian Key Laboratory of Laboratory Medicine, The First Affiliated Hospital of Fujian Medical University, Fuzhou, 350005 China

**Keywords:** Diagnostic biomarkers, Gut microbiota, Hepatocellular carcinoma, Liver cirrhosis, Reproducible genera

## Abstract

Hepatocellular carcinoma (HCC) is a malignant tumor with high incidence in China, which is mainly related to chronic hepatitis B (CHB) and liver cirrhosis (LC) caused by hepatitis B virus (HBV) infection. This study aimed to identify reproducible gut microbial biomarkers across Chinese population for LC and HCC diagnosis. In this study, a group of 21 CHB, 25 LC, 21 HCC and 15 healthy control (HC) were examined, and used as the training data. Four published faecal datasets from different regions of China were collected, totally including 121 CHB, 33 LC, 70 HCC and 96 HC. Beta diversity showed that the distribution of community structure in CHB, LC, HCC was significantly different from HC. Correspondingly, 14 and 10 reproducible differential genera across datasets were identified in LC and HCC, respectively, defined as LC-associated and HCC-associated genera. Two random forest (RF) models based on these reproducible genera distinguished LC or HCC from HC with an area under the curve (AUC) of 0.824 and 0.902 in the training dataset, respectively, and achieved cross-region validations. Moreover, AUCs were greatly improved when clinical factors were added. A reconstructed random forest model on eight genera with significant changes between HCC and non-HCC can accurately distinguished HCC from LC. Conclusively, two RF models based on 14 reproducible LC-associated and 10 reproducible HCC-associated genera were constructed for LC and HCC diagnosis, which is of great significance to assist clinical early diagnosis.

**Supplementary Information:**

The online version contains supplementary material available at 10.1186/s13568-023-01539-6.

## Key points

- Gut microbial biomarkers across Chinese population can be used as a non-invasive tool for the diagnosis of LC and HCC
- We constructed two RFmodels based on reproducible genera to distinguish LC or HCC from HC
- The efficacy of two models was greatly improved when clinical factors were added

**Supplementary Information:**

The online version contains supplementary material available at 10.1186/s13568-023-01539-6.

## Introduction

Hepatocellular carcinoma (HCC), the predominant form of liver cancer, is the third leading cause of cancer-related deaths worldwide. According to the statistics of the World Health Organization’s International Agency for Research on Cancer in 2020, there are about 410,000 new cases of HCC and 390,000 deaths in China. Its high prevalence, high mortality, and poor prognosis have led to serious public health problems. Different from the developed nations, the dominant reasons for the high incidence of HCC in China are chronic hepatitis B (CHB) resulting from hepatitis B virus (HBV) persistent infection, and HBV-induced liver cirrhosis (LC) (Chen et al. 2016). Patients with CHB are at high risk for progression to LC and eventually to HCC (Tu et al. 2014). About 70–90% of HCC patients are associated with cirrhosis (Lavanchy 2004). Early detection of precancerous cirrhosis and HCC can effectively improve the clinical outcome. However, due to the atypical symptom of early HCC, only about 30% of HCC are diagnosed at an early stage (Kudo 2012). The lack of methods for early diagnosis contributes to the urgency to develop novel biomarkers for LC and HCC.

The gut microbiome is the largest microbiome living in the human body. Relevant studies have reported that the gut microbiota plays a crucial role in liver disease (Chassaing et al. 2014). Xie et al. found that bile acid dysregulation caused by intestinal flora dysregulation was associated with the progression of liver disease to HCC (Xie et al. 2016). The bacteria and their products in the intestine can be transported to the liver through the gut-liver axis, which further promotes the occurrence of HCC (Dapito et al. 2012; Li et al. 2016; Yu et al. 2010). Many studies have reported that gut microbial markers are used as non-invasive diagnostic tools in type 2 diabetes (Qin et al. 2012), colorectal cancer (Yu et al. 2017), and pancreatic cancer (Ren et al. 2017). These studies provide a strong theoretical basis for intestinal microbes as a non-invasive tool for the early diagnosis of LC and HCC.

However, gut microbes are susceptible to the geographical environment, dietary habits, and technical differences. The gut microbial composition in samples collected from different regions was significant different (Rehman et al. 2016; Wilson et al. 2020; Yatsunenko et al. 2012), which lead to inconsistent results across studies. Moreover, there are few reports on the relationship between gut microbial alterations and the progression of HBV-induced liver diseases from CHB to LC and HCC.

This study aimed to explore the alterations of gut microbiota during the progression from healthy control (HC) to CHB, LC, and HCC, and develop reproducible gut microbial biomarkers for diagnosis of LC and HCC across Chinese population. A total of 82 stool samples from HBV-induced CHB, LC, HCC, and HC were collected and subjected to 16S rRNA gene sequencing. They were analyzed together with 320 samples (including 121 CHB, 33 LC, 70 HCC and 96 HC) in four public datasets from different regions of China. Compared with HC, reproducible differential genera across datasets were identified in LC and HCC, respectively. Two random forest (RF) classifier models based on these reproducible microbial biomarkers were constructed to distinguish LC or HCC from HC and verified in cross-region datasets. Furthermore, clinical indicators were added into the models to improve the diagnostic efficiency. This study highlighted the potential of the gut microbiota biomarkers as cross-region diagnostic tools for LC and HCC.

## Materials and methods

### Patient recruitment and stool sample collection

The study was approved by Ethics Review from Branch from Research and Clinical Technology Application, Ethics Committee of First Affiliated Hospital of Fujian Medical University (Approval No. MRCTA, ECFAH of FMU [2017]019) and performed according to the Helsinki Declaration. All participant signed informed consent before enrolment.

Patients who were diagnosed as chronic HBV liver disease with positive HBV surface antigen test for at least 6 months were recruited from the liver disease center of the first affiliated hospital of Fujian medical university. All participants were excluded from liver diseases caused by other viruses and alcohol, and the interference of other cancers and chronic diseases. In addition, the control group were healthy volunteers or healthy postgraduates of Fujian medical university. Finally, 82 samples, including 21 CHB patients, 25 LC patients, 21 HCC patients and 15 HC, were included and subjected to 16S rDNA gene sequencing. The V3-V4 hypervariable region of the bacterial 16S rDNA gene was amplified from the DNA samples with the barcoded forward primers (5′‐CTTTCCCTACACGAC‐3′) and reverse primers (5′‐ TGGAGTTCAGACGTGT‐3′). More detailed information can be found in our previous work (Zeng et al. 2020).Raw Illumina read data of this study were deposited in The National Genomics Data Center (NGDC) GSA (https://ngdc.cncb.ac.cn/gsa/) with accession number: CRA007561.

At the same time, 14 clinical indicators, including gender, age, body mass index (BMI), prothrombin time (PT), platelet count (PC), total bilirubin (TB), total protein (TP), alanine aminotransferase (ALT), aspartate aminotransferase (AST), alkaline phosphatase (AKP), triglycerides (TG), high-density lipoprotein (HDL), low-density lipoprotein (LDL), and alpha-fetoprotein (AFP), were collected (Additional file 1: Table S1).

### Public data collection

We searched for gut microbial studies from PubMed using the terms “chronic hepatitis B”, “liver cirrhosis” and “hepatocellular carcinoma”. The final inclusion conditions were: (1) patients in China; (2) 16S rRNA gene sequencing; (3) patients with liver diseases caused by HBV infection; (4) stool samples; (5) sequencing data and related sample information are publicly available. Finally, a total of 70 HCC, 33 LC, 121 CHB and 96 HC in four studies from Jilin (Northeast of China), Xiamen (Southeast of China), Nanjing and Shanghai (East of China) were included. Only clinical information in Jilin samples were available, including age, BMI, TP, ALT, AST, GGT, AFP, TB and albumin. Detailed information of datasets used in this study was shown in Table 1.

**Table 1:** Description of data used in this study

Data sources	HC	CHB	LC	HCC	Sequence region	Layout	No. of reads	City	Region of China
CRA007561	15	21	25	21	V3-V4	Paired	7.4 × 10^6^	Fuzhou	Southeast
SRP194355	20	8	8	35	V4	Paired	6.3 × 10^6^	Jilin	Northeast
SRP217171	21	28	25	–	V3-V4	Paired	9.8 × 10^6^	Xiamen	Southeast
SRP128442	33	–	–	35	V4	Single	4.8 × 10^6^	Nanjing	East
SRP103896	22	85	–	–	V3-V4	Paired	2.0 × 10^6^	Shanghai	East

### Unified data processing

Raw fastq files were downloaded from the sequence read archive (SRA) database. The quantitative insights into microbial ecology platform 2 (QIIME2) (Caporaso et al. 2010) was used to process all the raw sequencing data in a pipeline to obtain annotation profiles of taxis classification. All sample sequences were preprocessed using the same process as follows. The default parameters of FLASH software were used to splice the pair-ended paired samples, and other parameters were adjusted to –× 0.2; V3-V4 -M 200; V4 -M 150. The sequences with a quality score lower than 25 were filtered and the high-quality sequences were retained. Operational taxonomic unit (OTU) with 97% similarity was obtained by de novo clustering in each individual study. Then, chimera and monomer sequences were removed. The representative sequences of OTU were aligned to the SILVA (Quast et al. 2013) database for bacterial taxis classification, and the abundance profiles of bacterial classification at the phyla and genus levels were extracted for analysis.

### Statistical analysis

The Shannon index, Simpson index, Chao1 index and ACE index of alpha diversity were calculated by the “vegan” R package (Oksanen et al. 2020), and the differences between groups were compared by Kruskal–Wallis test. Beta diversity was measured by Bray–Curtis distance and the differences between groups were compared by permutational analysis of variance (PERMANOVA) with 999 randomized permutations. Principal coordinate analysis (PCoA) was used to display the beta diversity and the distribution between datasets. The microbial composition in each disease stage was analyzed at the phylum and genus levels, and the average relative abundance of each microbiota was calculated. Wilcoxon rank-sum test and Kruskal–Wallis rank-sum test was used to identify the microbiota with significant difference in HCC, LC and CHB compared with HC. All clinical indicators were tested by Kruskal–Wallis test except the chi-square test for gender. Spearman rank correlation was used to calculate the relationship between microbial makers and clinical indicators. Statistical significance was defined as *p* < 0.05.

### Model construction

A genus that was significantly different between HCC or LC and HC in two datasets and had the same dysregulation trend in the third dataset was defined as reproducible differential genus. RF models based on the reproducible differential genera were constructed to discriminate LC or HCC from HC. Five-fold cross-validation was performed to determine the optimal set of two parameters mtry and ntree, and the out-of-bag error rate was taken as a reference. The receiver operator characteristic (ROC) curve was plotted and the area under the curve (AUC) value were calculated to evaluate the effectiveness of the models. All statistical analyses were performed in R (version 3.6.1) software (https://cran.r-project.org/bin/windows/base/old/3.6.1/) (Dessau and Pipper 2008).

## Results

### Clinical characteristics of the patients and healthy individuals

As shown in Table 2, except for gender and BMI, other clinical characteristics of the participants in Fuzhou cohort were significantly different among disease states. In addition, age, TP, AST, and AFP were also significantly different between the Jilin and Fuzhou cohort (Additional file 1: Table S2). These results indicated that the HCC diagnostic biomarkers derived from these data ought to be independent of clinical characteristics.

**Table 2:** Clinical characteristics of 82 samples collected in this study

	Disease state (number of samples)				Statistic	*P* value
	HC (n = 15)	CHB (n = 21)	LC (n = 25)	HCC (n = 21)		
Gender(M/F)	10/5	14/7	19/6	18/3	2.575	0.462
Age	30.8 ± 6.21	40.14 ± 8.67	48.4 ± 13.16	53.14 ± 10.3	33.69	2.30E-07
BMI	21.55 ± 1.9	21.33 ± 3	22.52 ± 2.95	22.58 ± 3.14	3.29	0.349
PC	232.13 ± 31.28	158.38 ± 52.3	117.6 ± 94.38	121.76 ± 103.17	30.30	1.19E-06
PT	11.49 ± 0.79	13.41 ± 2	14.16 ± 3.82	15.07 ± 3.51	30.52	1.07E-06
TB	12.47 ± 3.39	57.55 ± 60.01	42.24 ± 66.39	22.67 ± 11.64	24.69	1.79E-05
TP	75.26 ± 4.91	69.31 ± 6.65	65.44 ± 7.82	63.5 ± 10.31	21.78	7.24E-05
ALT	19.13 ± 7.03	59.86 ± 29.39	53.6 ± 44.17	45.86 ± 45.26	23.87	2.65E-05
AST	21.67 ± 5.26	62 ± 28.94	70.76 ± 77.31	60.33 ± 57.8	31.17	7.83E-07
AKP	60.2 ± 16.51	96.62 ± 32.87	121 ± 61.13	139.57 ± 148.27	15.09	1.74E-03
AFP	2.19 ± 0.79	71.25 ± 135.66	957.86 ± 4344.36	511.62 ± 888.28	16.39	9.42E-04
TG	4.25 ± 0.58	1.25 ± 0.5	1.11 ± 0.75	0.96 ± 0.69	38.98	1.75E-08
HDL	1.49 ± 0.28	1 ± 0.52	0.87 ± 0.4	1.07 ± 0.42	19.61	2.04E-04
LDL	2.99 ± 0.51	2.18 ± 0.69	2.03 ± 0.82	2.66 ± 1.31	16.24	1.01E-03

Continuous variables were expressed as mean ± standard deviation. Chi-square test was used for gender, and Kruskal–Wallis test was used for other clinical variables

*NA* indicated not applicable

### Microbial diversity differences

**Fig. 1:**
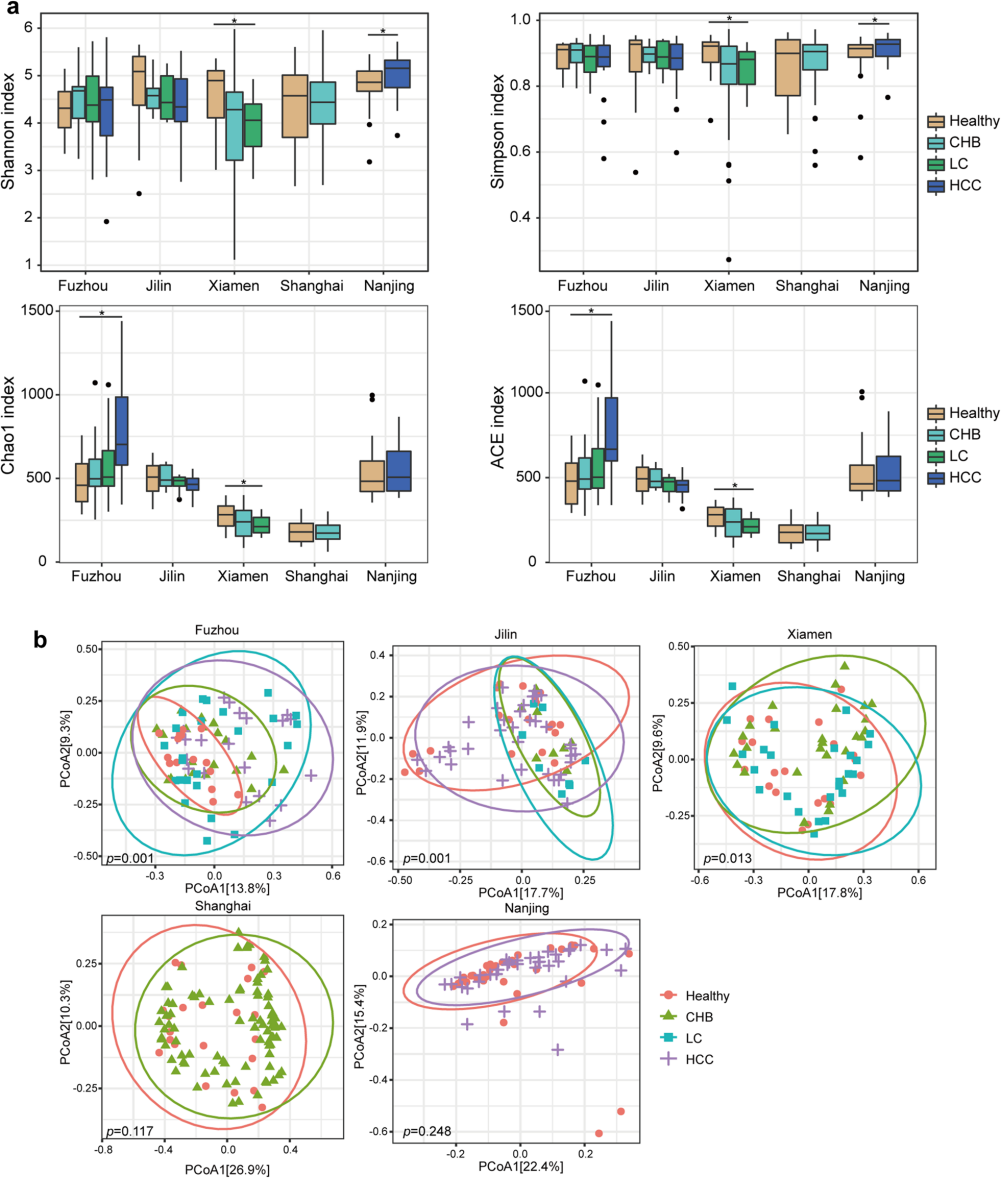
Microbial diversity differences between different groups. **a** Alpha diversity measured by the Shannon index, Simpson index, Chao1 index and ACE index. *: *p* < 0.05. **b** PCoA of beta diversity based on Bray–Curtis distance for five datasets

Firstly, we compared the microbial diversity of samples at various stages of liver disease with HC. The Shannon index, Simpson index, Chao1 index and ACE index of alpha diversity were calculated, respectively. The Kruskal–Wallis test showed that only the Shannon diversity in Fuzhou HCC samples were significantly higher than that in HC, and the Shannon, Chao1 and ACE diversity in Xiamen LC samples were significantly lower than that in HC (Kruskal–Wallis test, *p* < 0.05, Fig. 1a and Additional file 1: Tables S3, S4, S5, S6, S7). Notably, in three datasets with multiple disease stages, only the microbial diversity in the Xiamen samples was significantly decreased with disease progression.

Beta diversity was calculated using Bray–Curtis distance, and PCoA analysis showed that the compositions of individual microbial community structure among CHB, LC, HCC and HC were significantly different in Fuzhou, Jilin and Xiamen samples (Fig. 1b). The PERMANOVA results showed that disease stage (LC and HCC) exerted significant influences on the communities (Table 3), while CHB did not. Significant differences of beta diversity between CHB and HC were only observed in Fuzhou and Jilin samples but not in the Xiamen and Shanghai samples. The results indicated that the composition of the microbial community changed greatly in LC and HCC.

**Table 3:** PERMANOVA test results of beta diversity based on Bray–Curtis distance

	F-statistic	R^2^	*P-*value	Dataset
CHB vs HC	1.924	0.054	**0.006**	Fuzhou
CHB vs HC	2.809	0.098	**0.007**	Jilin
CHB vs HC	1.064	0.022	0.315	Xiamen
CHB vs HC	1.472	0.014	0.117	Shanghai
LC vs HC	3.284	0.080	**0.001**	Fuzhou
LC vs HC	4.315	0.142	**0.001**	Jilin
LC vs HC	1.931	0.042	**0.011**	Xiamen
HCC vs HC	2.981	0.081	**0.002**	Fuzhou
HCC vs HC	2.764	0.050	**0.001**	Jilin
HCC vs HC	1.230	0.018	0.220	Nanjing

R^2^ indicated the percentage of explained variance occupying in the total by group parameters, *P*-value indicated the significance and bold fonts indicated statistically significant differences between groups

Moreover, all samples from five datasets were pooled together for PCoA analysis to evaluate the biological variations and technical differences in different datasets. As shown in Additional file 1: Fig. S1, samples tended to cluster together by different studies rather than by different disease states. These results indicated that the heterogeneity between datasets was greater than the difference between different disease states. Therefore, different datasets were analyzed separately in the subsequent analysis.

### Alterations in microbial composition

**Fig. 2:**
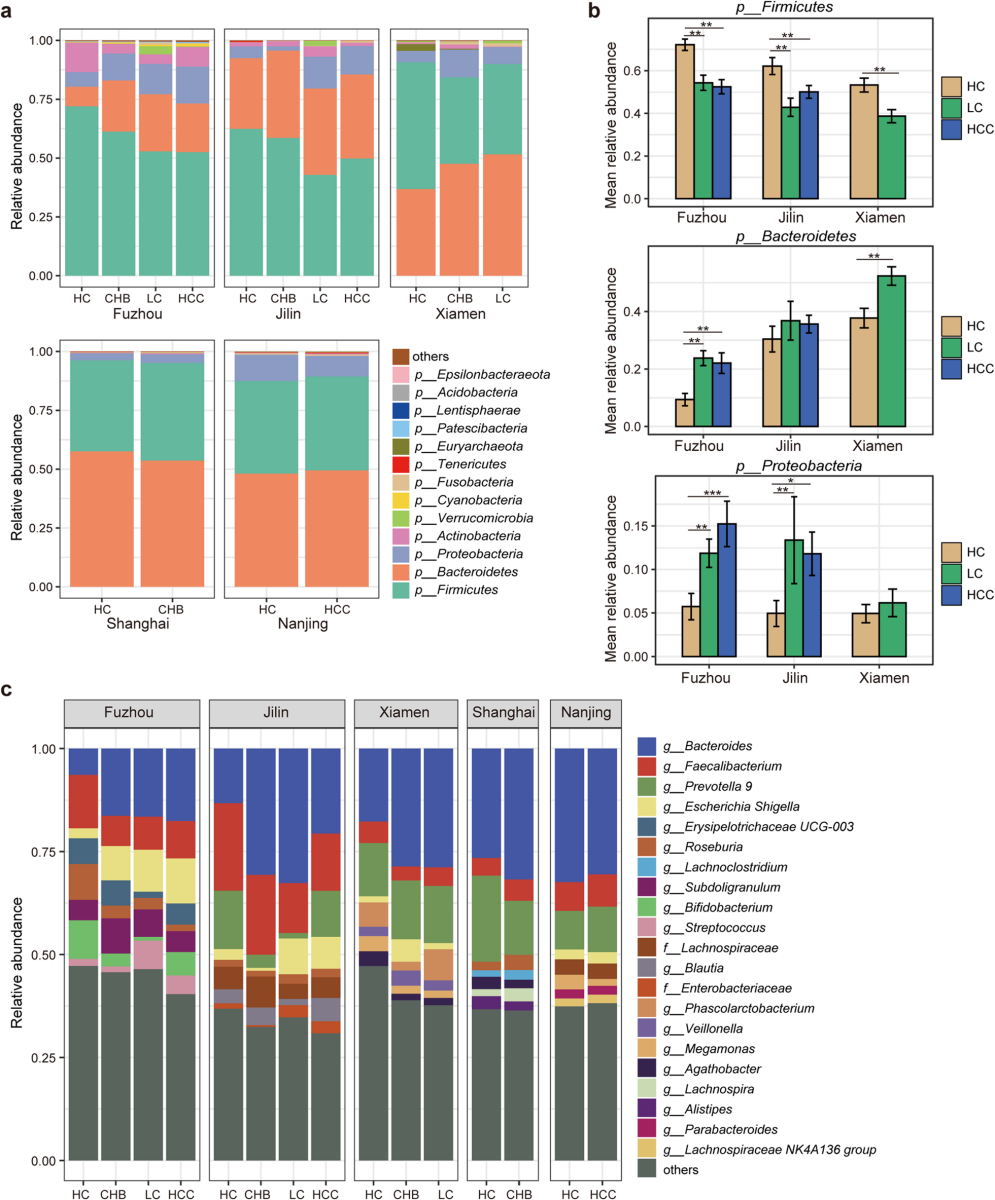
Distribution of the predominant bacteria at the phylum and genus levels in five datasets. **a** Stacked bars of the microbial composition at the phylum level among HC, CHB, LC and HCC. **b** Bar chart of the relative abundance of predominant taxa at the phylum levels in LC and HCC compare to HC. Wilcoxon rank sum test was used to compare the difference. *: *p* < 0.05, **:* p* < 0.01, ***:* p* < 0.001. **c** Stacked bars of the microbial composition at the genus level among HC, CHB, LC and HCC

In order to understand the specific changes of gut microbiota in different disease stages, we firstly analyzed the composition of gut microbiota at the phylum and genus levels. At the phylum level, *Firmicutes* and *Bacteroidetes* were the main dominant bacteria in HC, CHB, LC and HCC, followed by *Proteobacteria* and *Actinobacteria* (Fig. 2a). The relative abundances of *Firmicutes* in LC and HCC were significantly decreased compared to that in HC, and significantly decreased as disease progressed, while the relative abundance of *Bacteroides* was significantly increased (Wilcoxon rank-sum test, *p* < 0.05, Fig. 2b, Additional file 1: Fig S2a). Previous studies have shown that the ratio of *Bacteroidetes*/*Firmicutes* (B/F) is related to the development of inflammatory diseases, and the increase of the ratio can promote the development of inflammation (Kabeerdoss et al. 2015, Stojanov et al. 2020, Walker et al. 2011). The result indicated that patients with LC and HCC may be accompanied with more inflammatory responses. In addition, the relative abundance of *Proteobacteria* was also significantly increased in LC and HCC patients, suggesting that a high proportion of *Bacteroides/Firmicutes* and a high abundance of *Proteobacteria* may jointly contribute to the progression of HBV-induced liver disease (Fig. 2b).

**Fig. 3:**
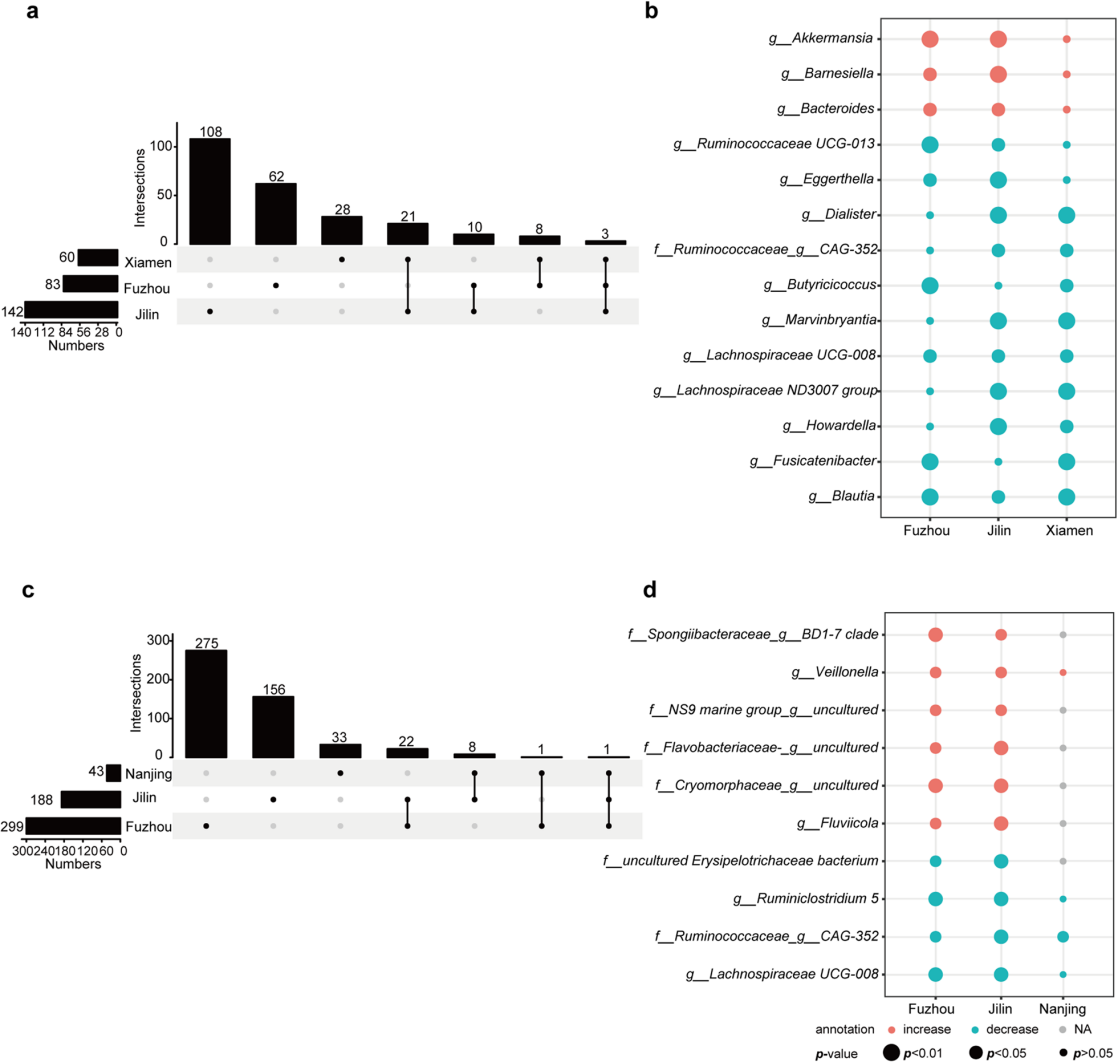
The significantly differential genera between LC or HCC and HC across datasets. **a**–**b** UpSet plot and bubble plot of the significantly differential genera between LC and HC across datasets. **c**–**d** UpSet plot and bubble plot of the significantly differential genera between HCC and HC across datasets. Red and green represented the direction of differential genera, the shape size represented the significant level. NA, not detected genera

At the genus level, the main bacteria composition were *Bacteroides*, *Faecalibacterium*, *Prevotella 9*, *Escherochia/Shigella*, *Erysipelotrichaceae UCG-003* and *Lachnoclostridium* (Fig. 2c). Compared with HC, 83, 142 and 60 differential genera were identified in Fuzhou, Jilin and Xiamen in LC samples, respectively (Wilcoxon rank-sum test, all *p* < 0.05, Fig. 3a), of which 14 genera were consistently dysregulated in at least two datasets, denoted as reproducible LC-associated microbial markers. Among the 14 genera, three genera (*Akkermansia, Barnesiella* and *Bacteroides*) were significantly increased in LC, while 11 genera (*Blautia, Fusicatenibacter, Howardella, Lachnospiraceae ND3007 Group, Lachnospiraceae UCG-008, Marvinbryantia, Butyricicoccus, CAG-352, Dialister, Eggerthella, Ruminococcaceae UCG-013*) were significantly decreased (*p* < 0.05, Fig. 3b). Similarly, 299, 188 and 43 genera with significant differences were identified between HCC and HC samples in Fuzhou, Jilin and Nanjing datasets (Wilcoxon rank-sum test, all *p* < 0.05, Fig. 3c), of which 10 genera were consistently dysregulated in at least two datasets, denoted as reproducible HCC-associated microbial markers. Among the 10 differential genera, six genera (*Fluviicola, Veillonella, Cryomorphaceae__uncultured, Flavobacteriaceae__uncultured, NS9 Marine group__uncultured bacterium, Spongiibacteraceae BD1-7 clade)* were significantly increased in HCC, while four genera (*Lachnospiraceae UCG-008, CAG-352, Ruminiclostridium 5, uncultured Erysipelotrichaceae bacterium*) were significantly decreased (*p* < 0.05, Fig. 3d).

In addition, the stepwise comparative analysis of CHB vs HC, LC vs CHB and HCC vs LC were also conducted, respectively. Compared with HC, 46, 130, 22 and 11 differential genera were identified in Fuzhou, Jilin, Xiamen and Nanjing CHB samples, respectively (Wilcoxon rank-sum test, all *p* < 0.05, Additional file 1: Fig S2b). Among them, *Bacteroides* was significantly increased in Fuzhou and Jilin datasets, while *Phascolarctobacterium*, *Gordonibacter* and *DTU089* were significantly decreased in Jilin and Nanjing datasets. Compared with CHB, there were 43, 92 and 48 differential genera in Fuzhou, Jilin and Xiamen LC samples, respectively (Wilcoxon Rank-sum test, all *p* < 0.05, Additional file 1: Fig S2c), of which 8 genera were consistently dysregulated in at least two datasets. Among them, *Bacteroides* was also significantly increased in two datasets, while 7 genera were significantly decreased. Compared with LC, 174 and 216 differential genera were identified in Fuzhou and Jilin HCC samples, respectively (Wilcoxon rank-sum test, all *p* < 0.05, Additional file 1: Fig S2d). Only 5 genera (*Ruminococcaceae UCG − 014*, *Akkermansia, Flavobacteriaceae__uncultured*, *Blautia* and *Eggerthella*) showed a consistent dysregulated direction, of which *Ruminococcaceae UCG − 014* and *Akkermansia* were significantly decreased.

### Construction the diagnostic model for LC on reproducible differential genera

**Fig. 4:**
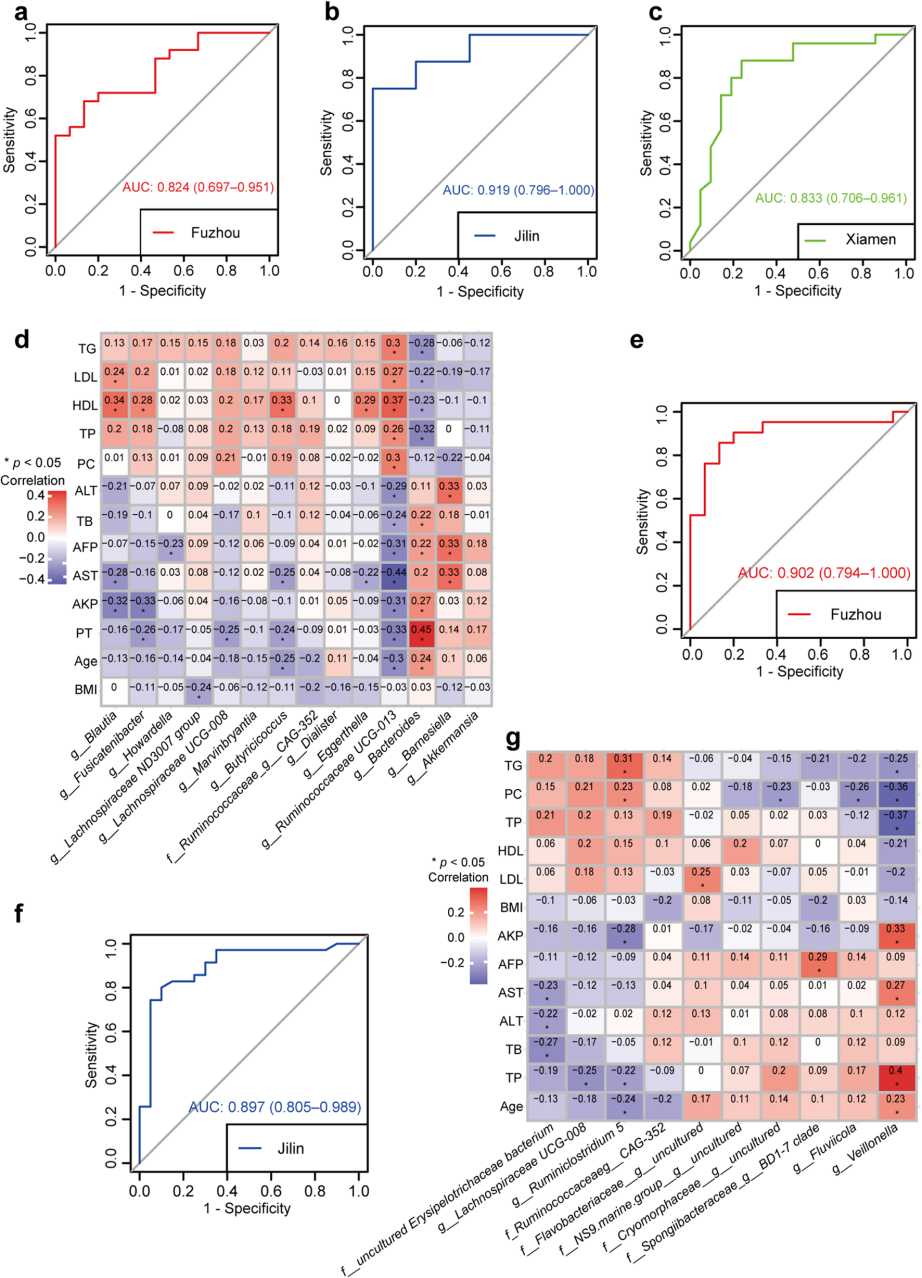
The performances of two RF models based on 14 LC-associated genera or 10 HCC-associated genera. **a**–**c** ROC curve of the RF model based on 14 LC-associated genera in Fuzhou, Jilin and Xiamen samples. **d** The heatmap of the relationships between 14 LC-associate genera and 13 clinical indicators. **e**–**f** ROC curve of the RF model based on 10 HCC-associated genera in Fuzhou and Jilin samples. **g** The heatmap of the relationships between 10 HCC-associated microbial genera and 13 clinical indicators

The following analysis was performed at the genus level. A RF classification model based on the 14 LC-associated genera was constructed to discriminate LC patients from HC. The Fuzhou samples were used as the training data and five-fold cross-validation was performed on a RF model with optimal parameter combination for mtry = 4 and ntree = 650. The AUC of the RF classifier model was 0.824 (95% CI 0.697–0.951, Fig. 4a) in Fuzhou samples. Then, the RF model achieved AUCs of 0.919 (95% CI 0.796–1.00, Fig. 4b) and 0.833 (95% CI 0.706–0.951, Fig. 4c) in Jilin and Xiamen samples, respectively. Moreover, AST to platelet ratio index (APRI), and FIB-4 are established as biomarkers for LC diagnosis in recent years, which were also applied in Fuzhou dataset with the same thresholds as previous studies (APRI: 1.5, FIB-4: 3.25) (Lurie et al. 2015; Xiao et al. 2015). The AUC values of APRI and FIB-4 for LC diagnosis were 0.72 and 0.51, respectively (Table 4), which were lower than the RF model based on 14 LC-associated genera. Collectively, these 14 LC-associated genera could be used as a potential microbial marker for LC diagnosis.

**Table 4:** Performance of conventional diagnostic biomarkers

Biomarkers	Fuzhou			Jilin		
	Sensitivity	Specificity	AUC	Sensitivity	Specificity	AUC
APRI	0.44	1.00	0.72	NA	NA	NA
FIB-4	0.56	0.47	0.51	NA	NA	NA
AFP	0.52	1.00	0.76	0.77	1.00	0.89

*NA* indicated not applicable

Correlation analysis between the above 14 common differential genera and 13 clinical factors in Fuzhou samples were performed. The results showed that 40 genera-factor pairs were significantly correlated, including 18 pairs with significantly positive correlation and 22 pairs with significantly negative correlation (Spearman, all *p* < 0.05, Fig. 4d). Among them, age, PT, AST, AKP, HDL and AFP were strongly correlated with the 14 LC-associated genera. In addition, *Ruminococcaceae UCG-013* was significant positively correlated with TG, LDL, HDL, TP and PC, and negatively correlated with age, PT, AKP, AFP and TB. *Bacteroides* was negatively correlated with TG, LDL, HDL and TP, and positively correlated with age, PT, AKP, AST, AFP, TB and ALT. Interestingly, the correlation relationship of *Ruminococcaceae UCG-013* and *Bacteroides* with clinical factors was opposite. Further correlation analysis showed that there was a marginally significant negative correlation between *Ruminococcaceae UCG-013* and *Bacteroides* (Spearman, R = − 0.2, *p* = 0.071).

To enhance the diagnostic efficacy for LC, clinical factors that were significantly correlated with the 14 microbial markers in Fuzhou samples and commonly collected in Jilin samples were selected as candidate features, including age, AST and AFP. Single or multiple clinical factors were added into the 14 LC-associated genera to reconstruct a classification model. The results showed that the classification accuracy of the reconstructed model was greatly improved (Additional file 1: Fig. S3a–f). The similar results were observed in Jilin cohort, which achieved the highest AUC combined age and AST. The results suggest that clinical factors (age, AST and AFP) can greatly improve the discrimination efficiency of the 14 LC-associated genera.

### Construction the diagnostic model for HCC on reproducible differential genera

Meanwhile, another RF classification model with optimal parameter combination for mtry = 9 and ntree = 200 by five-fold cross-validation was constructed based on the 10 HCC-associated genera to discriminate HCC from HC. The value of AUC in training Fuzhou samples was 0.902 (95% CI 0.794–1.00, Fig. 4e). Further, the model was validated in Jilin samples and achieved an AUC of 0.897 (95% CI 0.805–0.989, Fig. 4f). Validation was not performed in the Nanjing samples because only 4 of the 10 microbial markers were detected. Moreover, AFP is currently the most widely used biomarker for HCC diagnosis (Trevisani et al. 2001). As shown in Table 4, with the cut-off value of 10 ng/mL, the AUC values of AFP in differentiating HCC and HC were 0.76 in Fuzhou dataset and 0.89 in Jilin dataset, respectively, which were lower than the RF model based on 10 HCC-associated genera. These results indicated that the 10 HCC-associated genera could be used as potential microbial markers for HCC diagnosis. These results indicated that the classification efficiency of these 10 genera for HCC was better than the conventional diagnostic biomarker, and could be used as potential microbial markers for HCC diagnosis.

Correlation analysis between the above 10 genera and 13 clinical factors showed that 8 genera-clinical factor pairs were significant positively correlated and 12 genera-clinical factor pairs were significant negatively correlated (Spearman, all *p* < 0.05, Fig. 4g). Among them, *Veillonella* was significant positively correlated with age, PT, AST and AKP, and negatively correlated with TP, PC and TG. *Ruminiclostridium 5* was negatively correlated with age, PT and AKP, and positively correlated with PC and TG. The correlation between the two genera and clinical factors was opposite*.* Correlation analysis also demonstrated that the relative abundance of *Veillonella* was significant negatively correlated with that of *Ruminiclostridium 5* (Spearman, R = − 0.33, *p* = 0.0022).

Then single or multiple clinical factors, including age, AST and AFP, were combined with the 10 HCC-associated genera to reconstructed a model. The results showed that the classification accuracy was also greatly improved by the reconstructed model, which ranged from 0.921 to 0.990 (Additional file 1: Fig. S4a–f). The 10 microbial markers combined with AST and AFP achieved the highest AUCs in the two datasets (Additional file 1: Fig. S4f). These results indicated that clinical variables (age, AST and AFP) can greatly improve the ability of microbial markers to distinguish HCC patients.

### Identification the microbial markers for early diagnosis of HCC

**Fig. 5:**
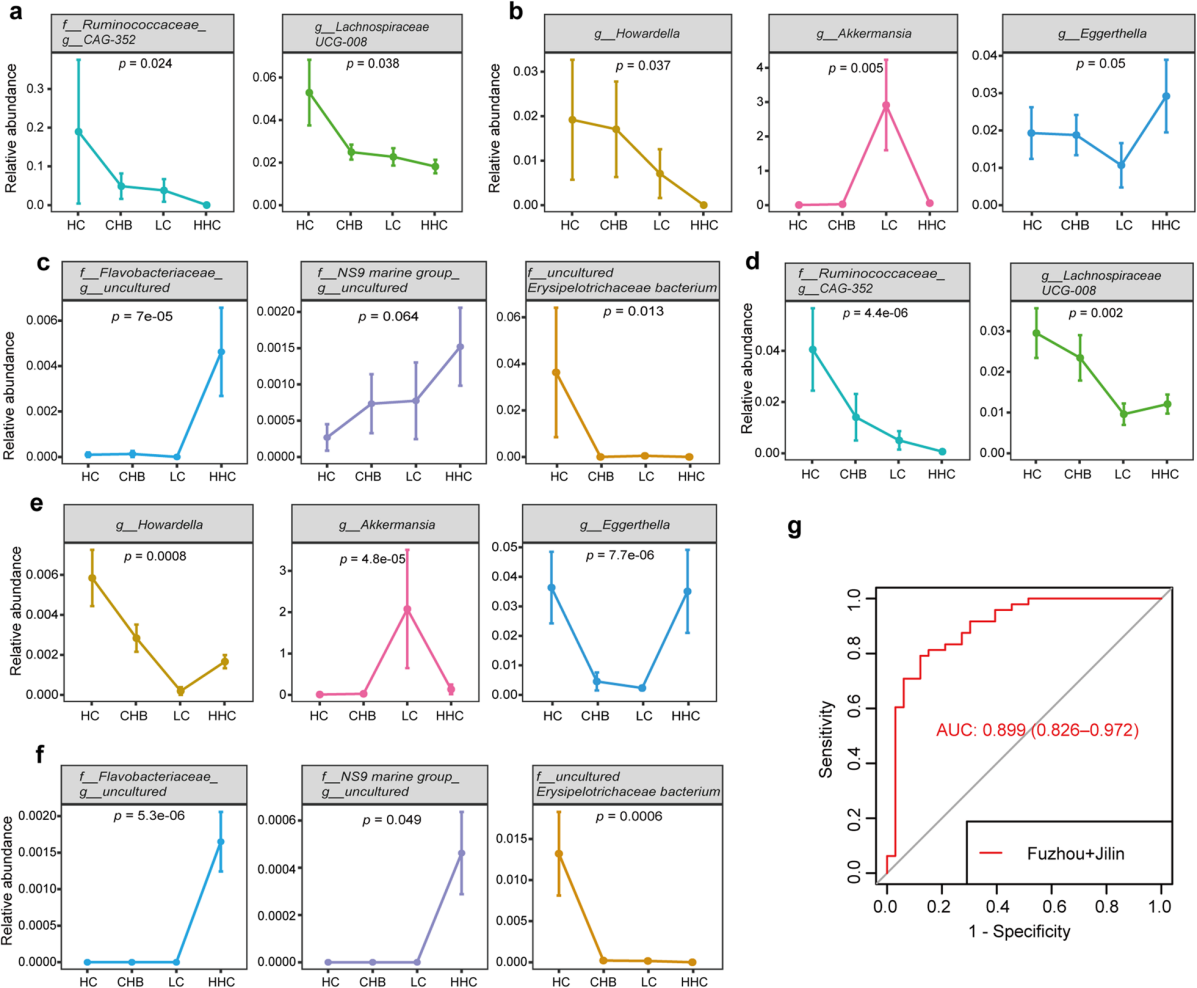
The alterations of microbial markers during disease progression. **a** Alterations of genera overlapped in the LC-associated genera and the HCC-associated genera in Fuzhou samples. **b** Alterations of LC-associated genera in Fuzhou samples. **c** Alterations of HCC-associated genera in Fuzhou samples. **d** Alterations of genera overlapped in the LC-associated genera and the HCC-associated genera in Jilin samples. **e** Alterations of LC-associated genera in Jilin samples. **f** Alterations of HCC-associated genera in Jilin samples. **g** ROC curve of the RF model based on eight genera for discriminating HCC and LC in the combined dataset of Fuzhou and Jilin samples

A multi-stage comparative analysis was performed in the 14 LC-associated genera and the 10 HCC-associated genera. In Fuzhou samples and Jilin samples, eight genera (*Ruminococcaceae__CAG-352, Howardella, Lachnospiraceae UCG-008, Akkermansia, Eggerthella, Flavobacteriaceae__uncultured, NS9 Marine group__uncultured bacterium, uncultured Erysipelotrichaceae bacterium*) were significantly different among multiple disease stages (Kruskal–Wallis test, *p* < 0.05). Among them, *Ruminococcaceae__CAG-352* and *Lachnospiraceae UCG-008* were shared by the LC-associated genera and the HCC-associated genera. In Fuzhou samples, the relative abundance of *Ruminococcaceae__CAG-352* sharply decreased from HC to CHB, LC and HCC, and the relative abundance of *Lachnospiraceae UCG-008* gradually decreased with the progression of disease (Fig. 5a). *Howardella, Akkermansia* and *Eggerthella* were unique in the LC-associated genera. The relative abundance of *Akkermansia* increased gradually in the precancerous stage of LC but decreased sharply in HCC, while the relative abundance of *Eggerthella* decreased gradually with the progression from HC to CHB and LC but increased significantly in HCC (Fig. 5b). Moreover, *Flavobacteriaceae__uncultured, NS9 Marine group_uncultured bacterium* and *uncultured Erysipelotrichaceae bacterium* were unique in the HCC-associated genera. The relative abundances of *Flavobacteriaceae__uncultured* and *NS9 Marine group uncultured bacterium* were very low in the precancerous samples, but increased sharply in Fuzhou HCC samples. The relative abundance of *uncultured Erysipelotrichaceae bacterium* was higher in HC, but significantly decreased or even disappeared in CHB, LC and HCC (Fig. 5c). The similar results were also observed in Jilin samples (Fig. 5d–f). These results suggested that the eight genera might play important roles in the progression from LC to HCC, which could be the potential microbial markers for the early diagnosis of HCC. Based on the above eight genera, a random forest classification model with optimal parameter combination for mtry = 6 and ntree = 2000 by five-fold cross-validation was constructed to distinguish HCC from LC by pooling Fuzhou and Jilin samples together. The model achieved an average AUC of 0.899 (95% CI 0.826–0.972, Fig. 5g), showing a good classification efficiency of HCC and LC.

## Discussion

This study comprehensively evaluated the alterations of gut microbiome in HBV-related liver diseases (including CHB, LC and HCC) across Chinese population, and identified 14 reproducible LC-associated genera and 10 reproducible HCC-associated genera. Two random forest classification models were developed based on these reproducible genera which accurately distinguished LC or HCC from HC and showed good diagnostic efficiencies in cross-region validation datasets. The diagnostic efficacy of the two set of microbial markers was further improved by combining relevant clinical variables (age, AST, AFP). In addition, a reconstructed random forest classification model based on eight genera which were significantly different among multiple disease stages had a good classification efficacy for HCC and LC. The results of this study suggested that gut microbial markers could be used as a promising non-invasive diagnostic tool for LC and HCC.

Previous studies have revealed that short chain fatty acid (SCFAs) was lower in stool samples from liver cirrhosis patients, and the abnormality became more obvious with the severity of liver disease, which may be an important factor promoting the development of liver cirrhosis (Jin et al. 2019). Notably, 10 of 14 LC-associated genera, including *Blautia, Fusicatenibacter, Howardella, Lachnospiraceae ND3007 Group, Lachnospiraceae UCG-008* and *Marvinbryantia* in the *Lachnospiraceae* family, and *Butyricicoccus, ruminococcaceae__CAG-352, Dialister, and Ruminococcaceae UCG-013* in the *Ruminococcaceae* family, which were closely related to the production of SCFAs, were decreased in LC samples. Another two increased genera, *Bacteroides* and *Barnesiella*, which belong to the *Bacteroidetes* family, are important clinical pathogens (Stojanov et al. 2020, Wexler 2007). These results showed that beneficial bacteria were significantly decreased and harmful bacteria was significantly increased in LC patients, which might corporately contribute to the progression of liver disease. Among the 10 reproducible microbial biomarkers identified at HCC, *Erysipelotrichaceae* has been found to be enriched in HCC (Pinero et al. 2019), *Veillonella* has been reported to be increased in LC and HCC groups (Tang et al. 2021).

Since both geographical location and lifestyle have significant impacts on human gut microbiota, cross-cohort studies provide the possibility to identify reproducible gut microbial biomarkers of cross-population. Several multi-cohort studies have combined metagenomic datasets to assess the diagnostic accuracy of gut microbiota in colorectal cancer across populations (Thomas et al. 2019; Wirbel et al. 2019). Recently, Ren et al. have established an early diagnostic model of HCC on 30 optimal operation taxa and validated in HCC samples from cross-region (Ren et al. 2019). However, they did not perform cross-region validation in LC samples. By integrating multiple cohort studies and using unified data preprocessing pipeline, this study identified reproducible LC-associated genera and HCC-associated genera, and constructed two random forest classification models to accurately distinguish LC or HCC from HC. Further, the altered gut microbiota from non-HCC samples to HCC samples highlighted the possibility of microbial markers to monitor and prevent HCC development.

However, the data collected from public database was very limited and the sample size for each disease stage was relatively small, especially the Jilin cohort contained only 8 cirrhosis samples with definite HBV infection. And the information of hepatitis B virus carrier was absence in all the datasets. In addition, due to the relatively low sequencing depth in Shanghai and Nanjing samples, the annotated microbiota and the common differential genera with other cohorts were relatively less. Moreover, this study included samples from Southeast, Northeast and East of China, but lacked samples from western China and central China. Therefore, more clinical data are still needed to validate and optimize the diagnostic models in this study. The stage information was unavailable and the efficacy of early diagnosis for HCC also need to be further evaluated. There are also obvious technical limitations. The data analyzed in this study were 16S rRNA gene sequencing data, which can only be annotated to the genus level. Further investigation of the bacterial species or functional gene families by metagenomic sequencing or integrated with multi-omics data may improve the diagnostic efficacy and help to understand the biological function.

In conclusion, this study revealed the alterations of gut microbiota in the progression of liver disease, and identified two list of reproducible microbial biomarkers that have the potential for non-invasive diagnosis for LC and HCC.

## Supplementary Information

**Additional file 1: Figure S1.** PCoA of samples from five datasets based on Bray-Curtis distance. PCoA analysis of samples from five datasets based on Bray–Curtis distance showed the fecal microbiota composition was different among studies (p < 0.01) and stages (p < 0.01). Datasets were color-coded and stages (HC, CHB, LC and CRC) were indicated by different shapes. **Figure S2.** The significantly differential microbe in the development of HCC. (a) Bubble plots of the significantly differential phyla of CHB vs HC, LC vs CHB and HCC vs LC across datasets. (b-d) UpSet plot and bubble plot of the significantly differential genera of CHB vs HC, LC vs CHB and HCC vs LC across datasets. Red and blue represented the direction of differential microbe, the shape size represented the significant level. **Figure S3.** ROC curve of the RF model based on 14 LC-associated genera combined with age, AST and AFP. (a-c) 14 LC-associated genera combined with age, AST and AFP, respectively. (d) 14 LC-associated genera combined with age and AST. (e) 14 LC-associated genera combined with age and AFP. (f) 14 LC-associated genera combined with AST and AFP. Figure S4. ROC curve of the RF model based on 10 HCC-associated genera combined with age, AST and AFP. (a-c) 10 HCC-associated genera combined with age, AST and AFP, respectively. (d) 10 HCC-associated genera combined with age and AST. (e) 10 HCC-associated combined with age and AFP. (f) 14 genera combined with AST and AFP. **Table S1.** The clinical indicators of Fuzhou samples. **Table S2.** Statistical analysis of clinical characteristics of patients in Jilin and Fuzhou datasets. **Table S3.** Alpha diversity in Fuzhou samples. **Table S4.** Alpha diversity in Jilin samples. **Table S5.** Alpha diversity in Xiamen samples. **Table S6.** Alpha diversity in Shanghai samples. **Table S7.** Alpha diversity in Nanjing samples.

## Data Availability

The dataset generated in this study, CRA007561, is restricted, but is available from the corresponding author on reasonable request. Publicly available datasets used in this study can be found in the SRA database with accession number: SRP194355, SRP217171, SRP128442 and SRP103896.
