# Supplementary material for: Identification reproducible microbiota biomarkers for the diagnosis of cirrhosis and hepatocellular carcinoma
Source: AMB Express. 2023 Mar 21;13:35. doi: 10.1186/s13568-023-01539-6 (PMC10030758; doi:10.1186/s13568-023-01539-6)
Supplement: Supplementary file 1 — Additional file 1: Figure S1. PCoA of samples from five datasets based on Bray-Curtis distance. PCoA analysis of samples from five datasets based on Bray–Curtis distance showed the fecal microbiota composition was different among studies (p < 0.01) and stages (p < 0.01). Datasets were color-coded and stages (HC, CHB, LC and CRC) were indicated by different shapes. Figure S2. The significantly differential microbe in the development of HCC. (a) Bubble plots of the significantly differential phyla of CHB vs HC, LC vs CHB and HCC vs LC across datasets. (b-d) UpSet plot and bubble plot of the significantly differential genera of CHB vs HC, LC vs CHB and HCC vs LC across datasets. Red and blue represented the direction of differential microbe, the shape size represented the significant level. Figure S3. ROC curve of the RF model based on 14 LC-associated genera combined with age, AST and AFP. (a-c) 14 LC-associated genera combined with age, AST and AFP, respectively. (d) 14 LC-associated genera combined with age and AST. (e) 14 LC-associated genera combined with age and AFP. (f) 14 LC-associated genera combined with AST and AFP. Figure S4. ROC curve of the RF model based on 10 HCC-associated genera combined with age, AST and AFP. (a-c) 10 HCC-associated genera combined with age, AST and AFP, respectively. (d) 10 HCC-associated genera combined with age and AST. (e) 10 HCC-associated combined with age and AFP. (f) 14 genera combined with AST and AFP. Table S1. The clinical indicators of Fuzhou samples. Table S2. Statistical analysis of clinical characteristics of patients in Jilin and Fuzhou datasets. Table S3. Alpha diversity in Fuzhou samples. Table S4. Alpha diversity in Jilin samples. Table S5. Alpha diversity in Xiamen samples. Table S6. Alpha diversity in Shanghai samples. Table S7. Alpha diversity in Nanjing samples. [file 13568_2023_1539_MOESM1_ESM.docx]

**AMB Express**

**Identification reproducible microbiota biomarkers for the diagnosis of cirrhosis and hepatocellular carcinoma**

Huarong Zhang^1†^, Junling Wu^2†^, Yijuan Liu^2,3†^, Yongbin Zeng^4,5^, Zhiyu Jiang^2^, Haidan Yan^1,2^, Jie lin^2^, Weixin Zhou^2^, Qishui Ou^4,5#^, Lu Ao^1,2,3#^

**Authors' Affiliations:**

^1^Key Laboratory of Ministry of Education for Gastrointestinal Cancer, the School of Basic Medical Sciences, Fujian Medical University, Fuzhou, 350122, China;

^2^Department of Bioinformatics, Fujian Key Laboratory of Medical Bioinformatics, School of Medical Technology and Engineering, Fujian Medical University, Fuzhou, China;

^3^ Department of Gastroenterology, The First Affiliated Hospital of Fujian Medical University, Fuzhou, China;

^4^Department of Laboratory Medicine, Gene Diagnosis Research Center, The First Affiliated Hospital of Fujian Medical University, Fuzhou, China；

^5^Fujian Key Laboratory of Laboratory Medicine, the First Affiliated Hospital of Fujian Medical University, Fuzhou, China.

† These authors contributed equally to this work and share first authorship.

**^#^Corresponding author:**

Lu Ao, Phone: +86-182-5905-6924, E-mail: [lukey@fjmu.edu.cn](mailto:lukey@fjmu.edu.cn)

Qishui Ou, Phone: +86-138-5013-6547, E-mail: [ouqishui@fjmu.edu.cn](mailto:ouqishui@fjmu.edu.cn)

Additional file **Figures**

**
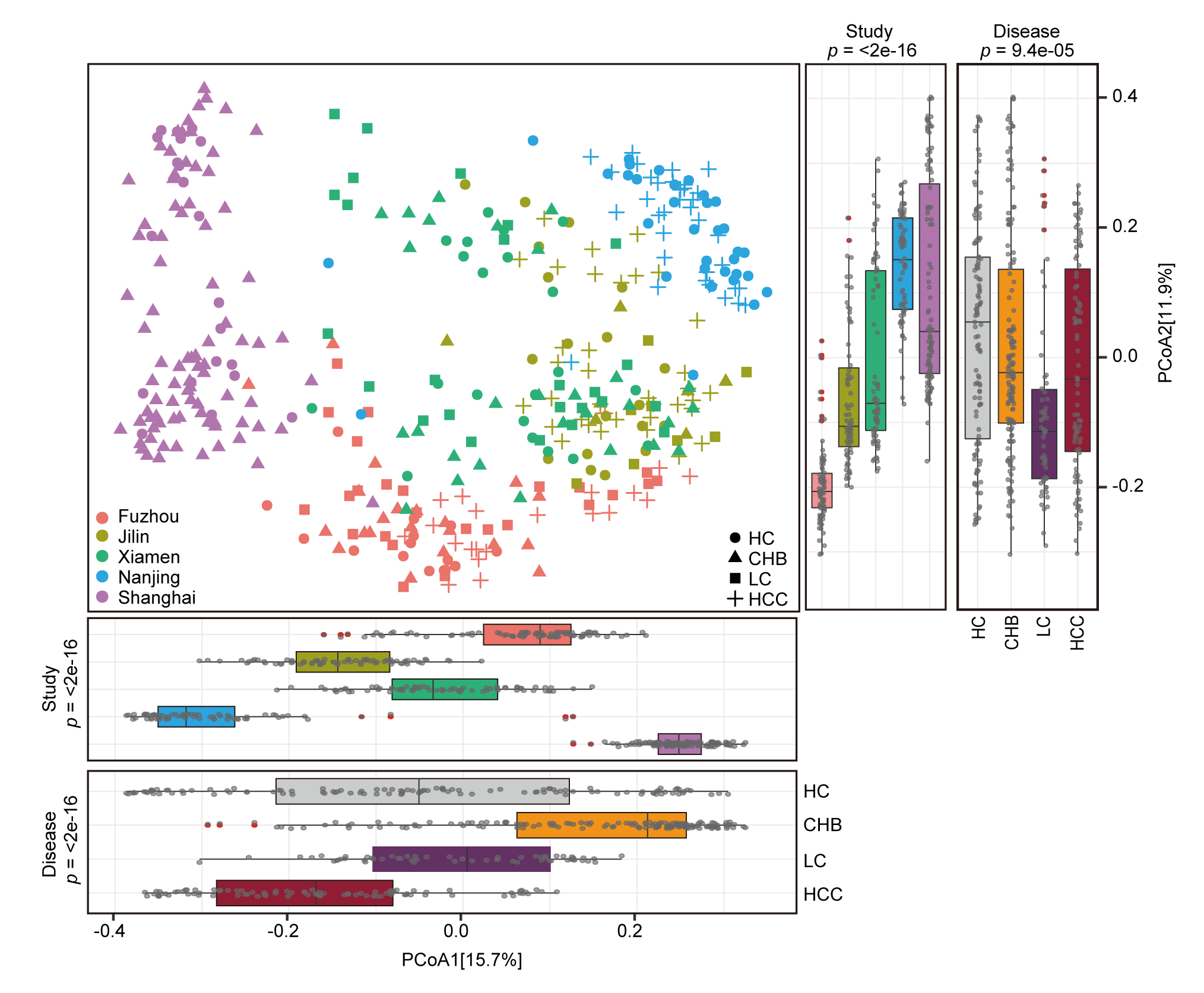
**

**Figure S1. PCoA of samples from five datasets based on Bray-Curtis distance**

PCoA analysis of samples from five datasets based on Bray–Curtis distance showed the fecal microbiota composition was different among studies (*p* < 0.01) and stages (*p* < 0.01). Datasets were color-coded and stages (HC, CHB, LC and CRC) were indicated by different shapes.


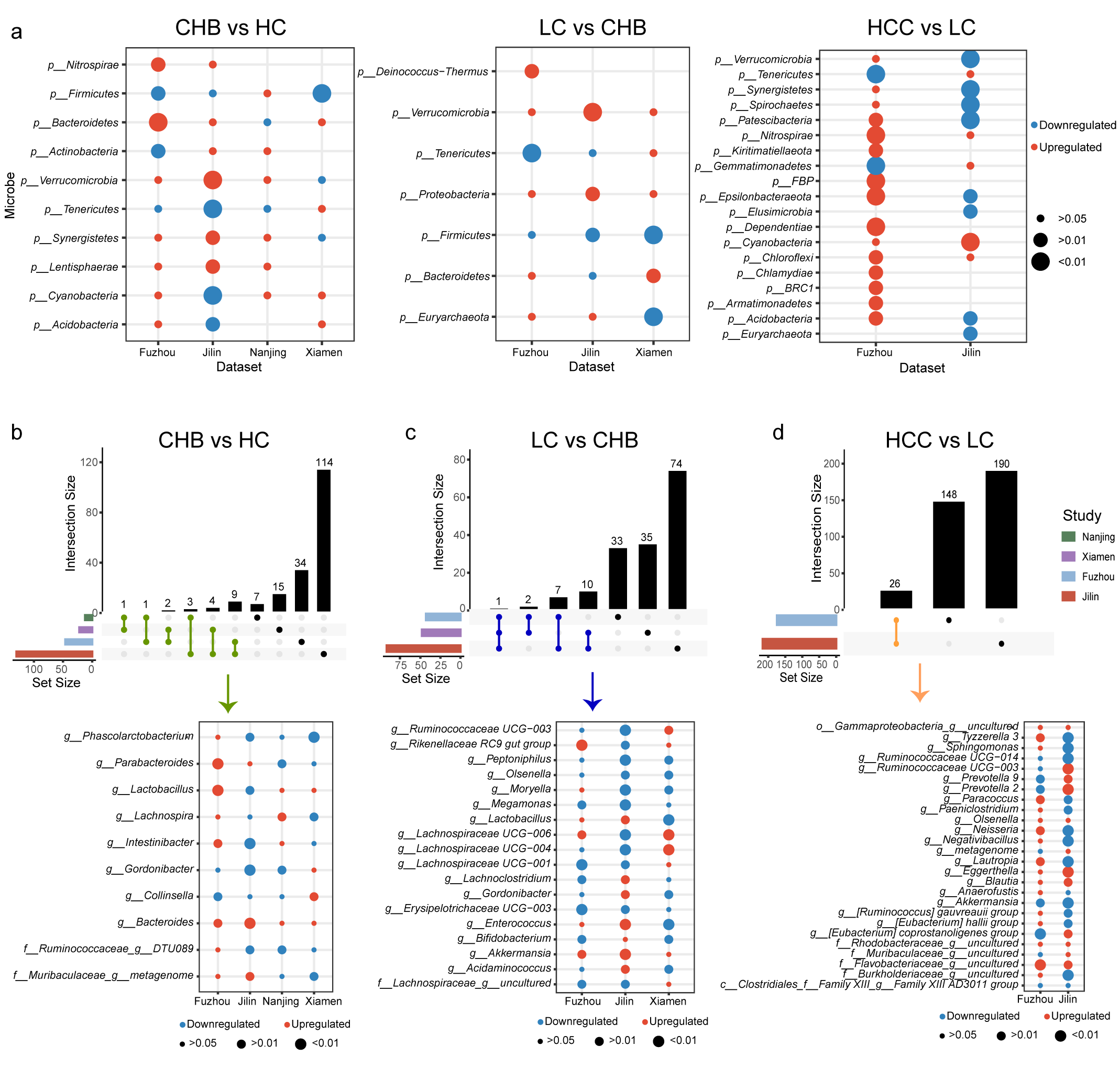


**Figure S2. The significantly differential microbe in the development of HCC**

(a) Bubble plots of the significantly differential phyla of CHB vs HC, LC vs CHB and HCC vs LC across datasets. (b-d) UpSet plot and bubble plot of the significantly differential genera of CHB vs HC, LC vs CHB and HCC vs LC across datasets. Red and blue represented the direction of differential microbe, the shape size represented the significant level.

**
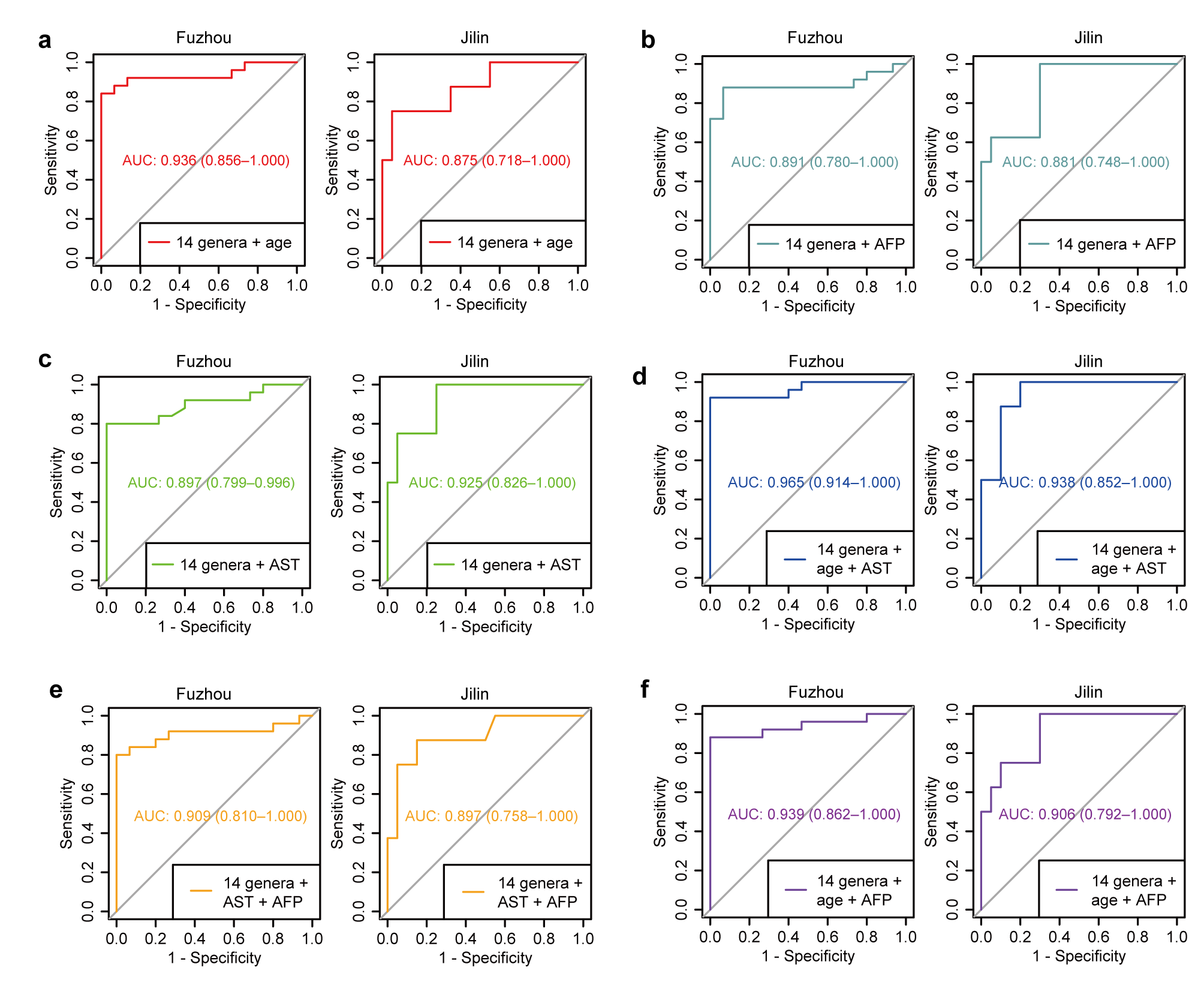
**

**Figure S3. ROC curve of the RF model based on 14 LC-associated genera combined with age, AST and AFP**

(a-c) 14 LC-associated genera combined with age, AST and AFP, respectively. (d) 14 LC-associated genera combined with age and AST. (e) 14 LC-associated genera combined with age and AFP. (f) 14 LC-associated genera combined with AST and AFP.

**
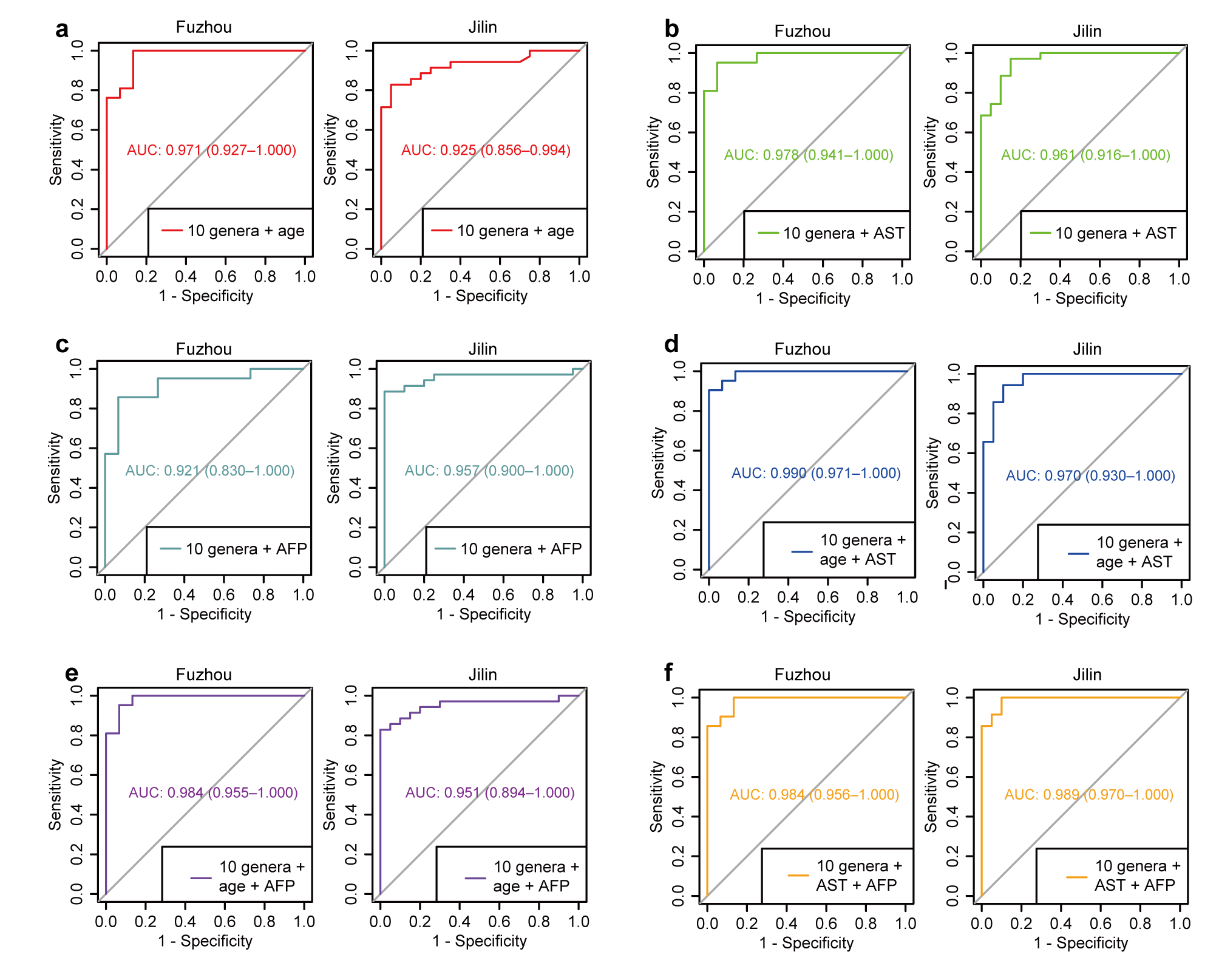
**

**Figure S4. ROC curve of the RF model based on 10 HCC-associated genera combined with age, AST and AFP**

1. c) 10 HCC-associated genera combined with age, AST and AFP, respectively. (d) 10 HCC-associated genera combined with age and AST. (e) 10 HCC-associated combined with age and AFP. (f) 14 genera combined with AST and AFP.

**Additional file Tables**

**Table S1. The clinical indicators of Fuzhou samples**

| Disease status | Age | BMI | PC | PT | TB | TP | ALT | AST | AKP | AFP | TG | HDL | LDL |
| --- | --- | --- | --- | --- | --- | --- | --- | --- | --- | --- | --- | --- | --- |
| HC | 27 | 19.88 | 254 | 11.8 | 10.7 | 66.8 | 13 | 15 | 47 | 1.77 | 3.53 | 1.42 | 3.12 |
| HC | 27 | 19.13 | 233 | 11.2 | 10 | 83.9 | 11 | 18 | 66 | 1.48 | 3.63 | 1.32 | 3.48 |
| HC | 30 | 18.29 | 284 | 12 | 11.4 | 73.5 | 12 | 19 | 68 | 2.4 | 5.1 | 1.61 | 3.31 |
| HC | 30 | 24.09 | 196 | 12.1 | 13.3 | 76.1 | 12 | 17 | 71 | 2.47 | 4.41 | 1.3 | 3.39 |
| HC | 26 | 20.98 | 218 | 10.6 | 13.6 | 73 | 24 | 18 | 63 | 1.13 | 3.84 | 1.58 | 2.95 |
| HC | 26 | 22.79 | 197 | 10.9 | 12.3 | 73.6 | 18 | 26 | 20 | 1.79 | 3.95 | 1.36 | 3.51 |
| HC | 35 | 21.72 | 170 | 11.7 | 17.9 | 70.6 | 14 | 19 | 62 | 4.28 | 3.61 | 1.15 | 3.14 |
| HC | 29 | 23.89 | 232 | 12.4 | 18.5 | 73.2 | 27 | 34 | 46 | 3.01 | 3.54 | 2.37 | 2.46 |
| HC | 38 | 20.2 | 227 | 12.3 | 7.7 | 77 | 25 | 24 | 67 | 1.44 | 4.46 | 1.48 | 3.22 |
| HC | 26 | 21.36 | 250 | 10.4 | 11.9 | 78.2 | 17 | 21 | 72 | 2.49 | 4.75 | 1.3 | 2.96 |
| HC | 28 | 22.65 | 241 | 10 | 18.6 | 70.1 | 30 | 27 | 92 | 2.51 | 4.26 | 1.53 | 1.96 |
| HC | 26 | 23.18 | 233 | 10.8 | 10.9 | 81 | 32 | 25 | 45 | 2.51 | 5.27 | 1.61 | 3.28 |
| HC | 33 | 19.44 | 230 | 11.7 | 9.5 | 80.3 | 13 | 18 | 64 | 1.45 | 4.94 | 1.46 | 2.71 |
| HC | 32 | 21.48 | 291 | 12.6 | 11.4 | 81.11 | 17 | 17 | 69 | 2.43 | 4.3 | 1.47 | 1.97 |
| HC | 49 | 24.24 | 226 | 11.8 | 9.4 | 70.5 | 22 | 27 | 51 | 1.71 | 4.16 | 1.4 | 3.43 |
| CHB | 60 | 23.52 | 88 | 15.3 | 53.5 | 68.8 | 55 | 22 | 143 | 10.6 | 0.7 | 0.64 | 2.31 |
| CHB | 45 | 23.42 | 127 | 12.3 | 31.1 | 76.4 | 72 | 67 | 96 | 42.77 | 0.95 | 1.77 | 2.54 |
| CHB | 29 | 18.07 | 126 | 12.5 | 23 | 64 | 17 | 26 | 44 | 2.08 | 0.59 | 1.52 | 0.45 |
| CHB | 27 | 20.11 | 269 | 13.3 | 43.6 | 70.3 | 82 | 76 | 99 | 0 | 1.31 | 0.61 | 2.32 |
| CHB | 38 | 17.47 | 134 | 12.6 | 22 | 60.7 | 48 | 53 | 76 | 1.48 | 1.26 | 1.79 | 1.09 |
| CHB | 38 | 24.96 | 180 | 12.6 | 86.8 | 82.5 | 81 | 79 | 143 | 5.56 | 1.78 | 0.55 | 2.63 |
| CHB | 40 | 26.12 | 202 | 14.6 | 48.2 | 58.7 | 45 | 65 | 73 | 3.83 | 1.88 | 0.64 | 2.51 |
| CHB | 34 | 19.53 | 146 | 14.6 | 55.8 | 77.8 | 22 | 36 | 121 | 31.17 | 0.9 | 0.89 | 2.55 |
| CHB | 33 | 17.34 | 123 | 12.8 | 27.5 | 64.9 | 51 | 58 | 90 | 6.36 | 0.57 | 0.88 | 2.22 |
| CHB | 51 | 17.8 | 58 | 12.7 | 46.8 | 63.9 | 93 | 101 | 142 | 6.35 | 1.46 | 1.34 | 1.57 |
| CHB | 48 | 19.03 | 190 | 12.6 | 25.7 | 79.5 | 92 | 94 | 134 | 33.12 | 0.91 | 1.77 | 3.2 |
| CHB | 56 | 21.85 | 137 | 11.7 | 24.9 | 77.6 | 32 | 29 | 80 | 4.24 | 0.92 | 1.12 | 2.11 |
| CHB | 39 | 24.16 | 170 | 12.7 | 16.5 | 62.9 | 53 | 52 | 70 | 62.04 | 1.16 | 1.25 | 2.02 |
| CHB | 37 | 23.53 | 195 | 12.8 | 22.4 | 74.1 | 54 | 41 | 89 | 23.4 | 0.93 | 1.12 | 2.51 |
| CHB | 34 | 19.05 | 223 | 11.4 | 131.7 | 67.3 | 77 | 76 | 124 | 459.8 | 2.39 | 0.43 | 2.18 |
| CHB | 41 | 23.66 | 168 | 12.8 | 17.5 | 68 | 68 | 96 | 59 | 1.23 | 1.26 | 1.19 | 3.8 |
| CHB | 45 | 17.91 | 123 | 12.9 | 18.4 | 61.5 | 24 | 27 | 58 | 2.28 | 1.12 | 1.29 | 2.05 |
| CHB | 43 | 24.01 | 138 | 20.5 | 222.3 | 69.9 | 141 | 132 | 114 | 191.9 | 1.87 | 0.11 | 1.6 |
| CHB | 32 | 19.53 | 90 | 16.1 | 211.4 | 66.5 | 65 | 59 | 82 | 434.5 | 1.73 | 0.1 | 1.84 |
| CHB | 43 | 26.23 | 216 | 11.7 | 68.1 | 67.7 | 60 | 77 | 142 | 170.6 | 1.88 | 0.48 | 2.03 |
| CHB | 30 | 20.56 | 223 | 13.2 | 11.3 | 72.5 | 25 | 36 | 50 | 3.04 | 0.72 | 1.47 | 2.32 |
| LC | 30 | 24.69 | 223 | 12.4 | 147.7 | 61.9 | 95 | 93 | 89 | 1210 | 3.68 | 0.61 | 1.8 |
| LC | 31 | 16.65 | 91 | 13.3 | 12.2 | 67.2 | 124 | 196 | 63 | 31.45 | 1.19 | 1.09 | 2.08 |
| LC | 39 | 22.1 | 25 | 18.7 | 34.5 | 56.2 | 29 | 40 | 133 | 2.9 | 0.47 | 0.45 | 0.9 |
| LC | 48 | 24.77 | 245 | 11.9 | 9.1 | 64.8 | 92 | 43 | 65 | 2.23 | 1.93 | 0.9 | 3.3 |
| LC | 45 | 20.9 | 164 | 12 | 16 | 66.9 | 42 | 41 | 171 | 2.54 | 0.95 | 1.41 | 2.99 |
| LC | 67 | 21.77 | 78 | 13.8 | 25.8 | 68 | 59 | 59 | 150 | 348.6 | 0.64 | 0.89 | 2.28 |
| LC | 50 | 22.06 | 384 | 14.6 | 14.8 | 79.3 | 13 | 42 | 73 | 2.71 | 1.2 | 0.58 | 1.58 |
| LC | 35 | 21.83 | 37 | 17.6 | 35 | 62.9 | 33 | 84 | 104 | 66.74 | 0.66 | 1.25 | 0.94 |
| LC | 64 | 20.34 | 78 | 13.8 | 11.6 | 65.6 | 14 | 33 | 77 | 10.7 | 0.81 | 0.35 | 2.01 |
| LC | 44 | 24.77 | 162 | 13.1 | 9.4 | 38 | 19 | 21 | 90 | 1.95 | 0.84 | 1.32 | 0 |
| LC | 67 | 22.22 | 87 | 15.9 | 25.2 | 66.7 | 66 | 76 | 169 | 13.19 | 0.56 | 0.84 | 1.94 |
| LC | 66 | 24 | 133 | 15 | 18.2 | 64.4 | 13 | 35 | 135 | 1.59 | 0.44 | 0.78 | 2.05 |
| LC | 67 | 22.22 | 53 | 17.3 | 25.2 | 66.7 | 66 | 116 | 169 | 13.19 | 0.56 | 0.84 | 1.94 |
| LC | 37 | 24.3 | 70 | 18.7 | 71.9 | 67.9 | 208 | 394 | 133 | 45.8 | 1.66 | 0.46 | 2.63 |
| LC | 42 | 24.91 | 33 | 16.9 | 31 | 67.5 | 27 | 44 | 206 | 8.77 | 0.64 | 1.34 | 1.72 |
| LC | 41 | 22.39 | 144 | 13.5 | 25.6 | 68.4 | 20 | 22 | 65 | 5.07 | 1.16 | 0.67 | 0.53 |
| LC | 37 | 23.99 | 38 | 20.6 | 326.3 | 67.7 | 91 | 86 | 196 | 46.91 | 1.94 | 0.06 | 1.99 |
| LC | 65 | 17.01 | 51 | 13.9 | 81.9 | 54.3 | 49 | 55 | 280 | 4.34 | 2.35 | 0.15 | 2.27 |
| LC | 35 | 28.4 | 73 | 16.4 | 29.2 | 75.3 | 39 | 40 | 60 | 4.7 | 0.67 | 0.86 | 1.25 |
| LC | 44 | 27.55 | 0 | 0 | 12.4 | 61.9 | 27 | 26 | 72 | 3.09 | 0.55 | 0.98 | 2.48 |
| LC | 37 | 23.67 | 191 | 11.5 | 9.3 | 71.4 | 23 | 25 | 55 | 1.41 | 0.39 | 1.75 | 2.47 |
| LC | 56 | 17.16 | 84 | 14 | 14.4 | 68.6 | 30 | 29 | 95 | 331.5 | 1.29 | 1.26 | 2.7 |
| LC | 37 | 24.41 | 106 | 12 | 16.2 | 75.3 | 71 | 72 | 50 | 4.32 | 1.55 | 0.91 | 2.79 |
| LC | 68 | 22.21 | 69 | 14 | 24.2 | 64.6 | 21 | 34 | 223 | 6.81 | 0.97 | 1.12 | 2.77 |
| LC | 58 | 18.78 | 321 | 13 | 28.8 | 64.4 | 69 | 63 | 102 | 21776 | 0.65 | 0.87 | 3.25 |
| HCC | 28 | 19.21 | 45 | 15.1 | 40 | 69.8 | 36 | 44 | 107 | 26.3 | 0.39 | 1.25 | 0.91 |
| HCC | 67 | 25.39 | 84 | 16.5 | 20.3 | 61.9 | 17 | 42 | 114 | 2.29 | 0.38 | 1.18 | 2.1 |
| HCC | 43 | 15.99 | 146 | 12.4 | 15.8 | 74.3 | 19 | 24 | 66 | 3578 | 0.63 | 1.34 | 2.71 |
| HCC | 42 | 25.04 | 183 | 12.6 | 18.4 | 73.2 | 27 | 28 | 58 | 178.5 | 0.93 | 0.93 | 2.98 |
| HCC | 51 | 21.64 | 89 | 25.7 | 20.7 | 64.9 | 23 | 37 | 90 | 88.68 | 0.55 | 1.87 | 2.94 |
| HCC | 58 | 27.64 | 61 | 14 | 43.6 | 62.5 | 29 | 30 | 59 | 1.8 | 0.63 | 0.95 | 1.4 |
| HCC | 43 | 22.31 | 184 | 12.9 | 17.4 | 72.8 | 60 | 53 | 79 | 1729 | 0.81 | 1.18 | 3.12 |
| HCC | 51 | 27.28 | 128 | 13.1 | 11.1 | 65.5 | 38 | 39 | 85 | 644.1 | 0.81 | 0.23 | 2.5 |
| HCC | 41 | 25.53 | 119 | 17.7 | 12.4 | 38.2 | 32 | 31 | 75 | 3.74 | 0.79 | 1.23 | 3.08 |
| HCC | 66 | 21.36 | 114 | 23 | 51 | 67.9 | 196 | 269 | 338 | 1210 | 0.55 | 0.75 | 1.65 |
| HCC | 65 | 23.61 | 163 | 12.5 | 29.5 | 52.7 | 155 | 142 | 43 | 2.54 | 1.13 | 0.86 | 2.7 |
| HCC | 48 | 20.31 | 95 | 15.3 | 28.4 | 66.6 | 51 | 144 | 204 | 1210 | 1.09 | 1.52 | 5.38 |
| HCC | 47 | 19.83 | 71 | 13.1 | 28.1 | 69.7 | 47 | 53 | 59 | 2.05 | 0.71 | 1.11 | 2.24 |
| HCC | 55 | 20.28 | 105 | 13.5 | 17.5 | 70.9 | 44 | 34 | 63 | 797.9 | 1 | 1.67 | 2.97 |
| HCC | 55 | 23.46 | 523 | 13 | 11.2 | 64.9 | 19 | 62 | 704 | 1210 | 3.71 | 0.68 | 6.49 |
| HCC | 57 | 26.54 | 87 | 13.7 | 7.4 | 36.2 | 15 | 32 | 145 | 2.36 | 0.61 | 1.07 | 1.56 |
| HCC | 62 | 18.83 | 41 | 15.2 | 16.1 | 60.9 | 28 | 35 | 137 | 1.63 | 0.81 | 1.07 | 2.04 |
| HCC | 67 | 25.82 | 10 | 17.8 | 24.3 | 55.3 | 24 | 32 | 243 | 41.37 | 1.37 | 0.62 | 0.87 |
| HCC | 61 | 20.83 | 59 | 13.6 | 12.5 | 68.2 | 23 | 54 | 130 | 9.66 | 0.98 | 1.69 | 3.23 |
| HCC | 49 | 23.11 | 93 | 13.5 | 34.2 | 70.3 | 49 | 44 | 85 | 1.37 | 1.35 | 0.98 | 2.4 |
| HCC | 60 | 20.1 | 157 | 12.3 | 16.1 | 66.7 | 31 | 38 | 47 | 2.66 | 0.89 | 0.35 | 2.55 |

BMI: body mass index, PC: platelet count, PT: prothrombin time, TB: total bilirubin, TP: total protein, ALT: alanine aminotransferase, AST: aspartate aminotransferase, AKP: alkaline phosphatase, AFP: alpha-fetoprotein, TG: triglycerides, HDL: high-density lipoprotein and LDL: low-density lipoprotein.

**Table S2 Statistical analysis of clinical characteristics of patients in Jilin and Fuzhou datasets**

| Characteristics | Dataset | Mean±SD | Statistic | *P* value |
| --- | --- | --- | --- | --- |
| Age | Fuzhou | 44.28±12.90 | 30.02 | 4.28E-08 |
|  | Jilin | 55.52±8.36 |  |  |
| BMI | Fuzhou | 22.05±2.86 | 2.10 | 0.15 |
|  | Jilin | 22.69±1.83 |  |  |
| TB | Fuzhou | 35.70±50.11 | 1.35 | 0.25 |
|  | Jilin | 22.44±15.21 |  |  |
| TP | Fuzhou | 67.73±8.77 | 4.12 | 0.04 |
|  | Jilin | 69.83±5.48 |  |  |
| ALT | Fuzhou | 46.91±38.81 | 1.39 | 0.24 |
|  | Jilin | 37.21±29.14 |  |  |
| AST | Fuzhou | 56.87±55.73 | 13.41 | 2.51E-04 |
|  | Jilin | 37.42±34.76 |  |  |
| AFP | Fuzhou | 441.70±2438.51 | 6.14 | 0.01 |
|  | Jilin | 4080.75±11829.31 |  |  |

Kruskal-Wallis test was used to calculate the *p* value**.**

**Table S3 Alpha diversity in Fuzhou samples**

|  | obs.dif | critical.dif | difference | p value | alpha diversity |
| --- | --- | --- | --- | --- | --- |
| CHB-HCC | 6.523809524 | 19.39000156 | FALSE | 0.811297315 | Simpson |
| CHB-HCC | 11.80952381 | 19.39000156 | FALSE | 0.374678626 | Chao |
| CHB-HCC | 15.0952381 | 19.39000156 | FALSE | 0.168483566 | Shannon |
| CHB-HCC | 15.0952381 | 19.39000156 | FALSE | 0.168483566 | ACE |
| CHB-HC | 10.34285714 | 21.24068249 | FALSE | 0.572717474 | Simpson |
| CHB-HC | 11.0952381 | 21.24068249 | FALSE | 0.513112807 | Chao |
| CHB-HC | 3.761904762 | 21.24068249 | FALSE | 0.966214522 | Shannon |
| CHB-HC | 8.542857143 | 21.24068249 | FALSE | 0.713249947 | ACE |
| CHB-LC | 2.44952381 | 18.59823614 | FALSE | 0.98559484 | Simpson |
| CHB-LC | 3.961904762 | 18.59823614 | FALSE | 0.943276055 | Chao |
| CHB-LC | 0.744761905 | 18.59823614 | FALSE | 0.999578229 | Shannon |
| CHB-LC | 6.16952381 | 18.59823614 | FALSE | 0.817735056 | ACE |
| HCC-LC | 16.53142857 | 18.59823614 | FALSE | 0.08805649 | Shannon |
| HCC-LC | 7.847619048 | 18.59823614 | FALSE | 0.681367503 | ACE |
| HCC-LC | 14.35047619 | 18.59823614 | FALSE | 0.174879328 | Simpson |
| HCC-LC | 13.17714286 | 18.59823614 | FALSE | 0.241354315 | Chao |
| HC-LC | 6.44 | 20.52044882 | FALSE | 0.841222379 | Shannon |
| HC-LC | 7.133333333 | 20.52044882 | FALSE | 0.795717094 | ACE |
| HC-LC | 4.506666667 | 20.52044882 | FALSE | 0.938294051 | Simpson |
| HC-LC | 5.613333333 | 20.52044882 | FALSE | 0.888489839 | Chao |
| HCC-HC | 22.97142857 | 21.24068249 | TRUE | **0.022472493** | Shannon |
| HCC-HC | 0.714285714 | 21.24068249 | FALSE | 0.999749916 | ACE |
| HCC-HC | 18.85714286 | 21.24068249 | FALSE | 0.088663574 | Simpson |
| HCC-HC | 18.79047619 | 21.24068249 | FALSE | 0.090435991 | Chao |

Obs.dif: observed difference; critical.dif: critical difference; difference indicates whether there is a significant difference between two groups. Kruskal-Wallis test was used to calculate the *p* value.

**Table S4 Alpha diversity in Jilin samples**

|  | obs.dif | critical.dif | difference | *p* value | alpha diversity |
| --- | --- | --- | --- | --- | --- |
| CHB-HCC | 9.457142857 | 21.33915194 | FALSE | 0.64632838 | Shannon |
| CHB-HCC | 4.067857143 | 21.33915194 | FALSE | 0.958404281 | Simpson |
| CHB-HCC | 12.65714286 | 21.33915194 | FALSE | 0.398857272 | Chao |
| CHB-HCC | 13.07857143 | 21.33915194 | FALSE | 0.368948416 | ACE |
| CHB-HC | 0.85 | 22.77933115 | FALSE | 0.999658561 | Shannon |
| CHB-HC | 2.525 | 22.77933115 | FALSE | 0.991299629 | Simpson |
| CHB-HC | 4.95 | 22.77933115 | FALSE | 0.940071917 | Chao |
| CHB-HC | 4.75 | 22.77933115 | FALSE | 0.946540374 | ACE |
| CHB-LC | 14 | 27.22650829 | FALSE | 0.526760175 | Shannon |
| CHB-LC | 7.375 | 27.22650829 | FALSE | 0.891331527 | Simpson |
| CHB-LC | 21 | 27.22650829 | FALSE | 0.175158388 | Chao |
| CHB-LC | 21.875 | 27.22650829 | FALSE | 0.146780325 | ACE |
| HCC-HC | 10.30714286 | 15.26350483 | FALSE | 0.282183502 | Shannon |
| HCC-HC | 6.592857143 | 15.26350483 | FALSE | 0.664978084 | Simpson |
| HCC-HC | 7.707142857 | 15.26350483 | FALSE | 0.542334677 | Chao |
| HCC-HC | 8.328571429 | 15.26350483 | FALSE | 0.474541669 | ACE |
| HCC-LC | 4.542857143 | 21.33915194 | FALSE | 0.943377367 | Shannon |
| HCC-LC | 3.307142857 | 21.33915194 | FALSE | 0.976930389 | Simpson |
| HCC-LC | 8.342857143 | 21.33915194 | FALSE | 0.730958508 | Chao |
| HCC-LC | 8.796428571 | 21.33915194 | FALSE | 0.697176913 | ACE |
| HC-LC | 14.85 | 22.77933115 | FALSE | 0.313210757 | Shannon |
| HC-LC | 9.9 | 22.77933115 | FALSE | 0.660569048 | Simpson |
| HC-LC | 16.05 | 22.77933115 | FALSE | 0.245975204 | Chao |
| HC-LC | 17.125 | 22.77933115 | FALSE | 0.194206013 | ACE |

**Table S5 Alpha diversity in Xiamen samples**

|  | obs.dif | critical.dif | difference | *p* value | alpha diversity |
| --- | --- | --- | --- | --- | --- |
| CHB-HC | 12.29761905 | 14.86228986 | FALSE | 0.11691775 | Shannon |
| CHB-HC | 11.63095238 | 14.86228986 | FALSE | 0.14639913 | Simpson |
| CHB-HC | 6.702380952 | 14.86228986 | FALSE | 0.526745956 | Chao |
| CHB-HC | 6.630952381 | 14.86228986 | FALSE | 0.533936535 | ACE |
| CHB-LC | 5.572857143 | 14.16658804 | FALSE | 0.613817385 | Shannon |
| CHB-LC | 2.052857143 | 14.16658804 | FALSE | 0.93581937 | Simpson |
| CHB-LC | 15.88428571 | 14.16658804 | TRUE | **0.019889612** | Chao |
| CHB-LC | 14.76714286 | 14.16658804 | TRUE | **0.033649746** | ACE |
| HC-LC | 17.87047619 | 15.2396718 | TRUE | **0.01384513** | Shannon |
| HC-LC | 13.68380952 | 15.2396718 | FALSE | 0.080130909 | Simpson |
| HC-LC | 22.58666667 | 15.2396718 | TRUE | **0.001129479** | Chao |
| HC-LC | 21.39809524 | 15.2396718 | TRUE | **0.002237498** | ACE |

**Table S6 Alpha diversity in Shanghai samples**

|  | obs.dif | critical.dif | difference | *p* value | alpha diversity |
| --- | --- | --- | --- | --- | --- |
| CHB-HC | 0.629411765 | 14.54897241 | FALSE | 0.932427414 | Shannon |
| CHB-HC | 3.662032086 | 14.54897241 | FALSE | 0.621779121 | Simpson |
| CHB-HC | 0.057219251 | 14.54897241 | FALSE | 0.993849737 | Chao |
| CHB-HC | 0.171657754 | 14.54897241 | FALSE | 0.981550672 | ACE |

**Table S7 Alpha diversity in Nanjing samples**

|  | obs.dif | critical.dif | difference | *p* value | alpha diversity |
| --- | --- | --- | --- | --- | --- |
| HCC-HC | 8.036363636 | 9.403725297 | FALSE | 0.093939585 | Shannon |
| HCC-HC | 6.976623377 | 9.403725297 | FALSE | 0.145919362 | Simpson |
| HCC-HC | 0.971428571 | 9.403725297 | FALSE | 0.839549912 | Chao1 |
| HCC-HC | 0.735930736 | 9.403725297 | FALSE | 0.878094058 | ACE |
